# Atherosclerosis at Warp Speed: The Urgent Lessons of Homozygous Familial Hypercholesterolemia

**DOI:** 10.1016/j.jaccas.2026.108238

**Published:** 2026-06-10

**Authors:** David Drossner

**Affiliations:** Department of Pediatric Cardiology, Joe Dimaggio Children's Hospital Memorial Healthcare System, Hollywood, Florida, USA

**Keywords:** lipid metabolism disorders, myocardial infarction, percutaneous coronary intervention

**Figure:**
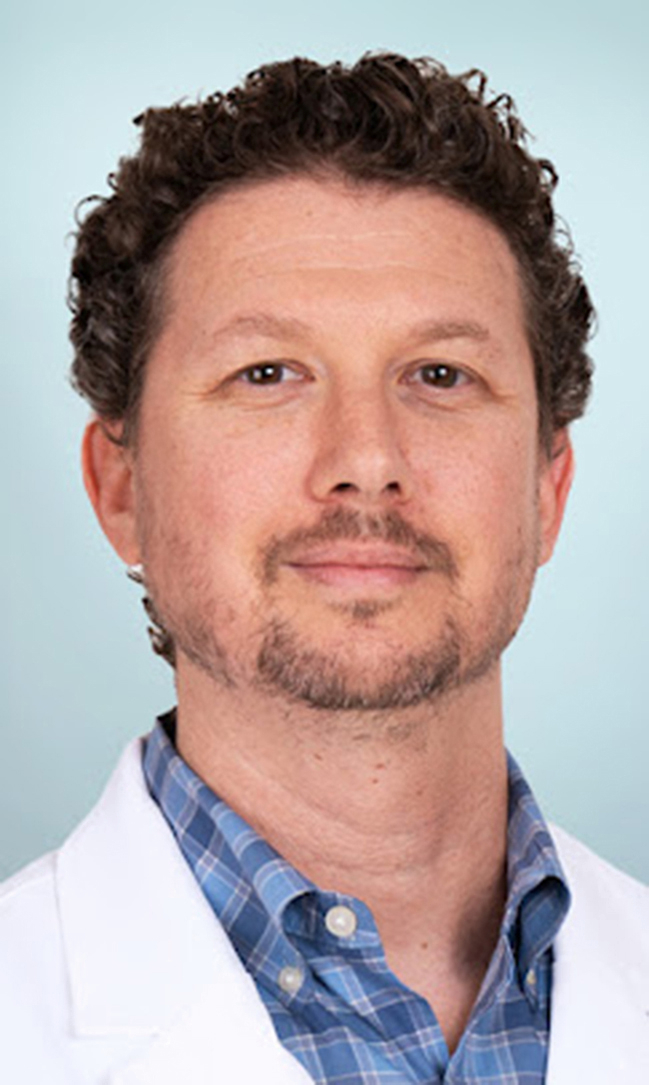

In this issue of *JACC: Case Reports*, Siddiqui et al^1^ present a 13-year-old boy with acute anterior ST-segment elevation myocardial infarction, cardiogenic shock, and severe multivessel coronary disease, vividly illustrating the devastating phenotypic expression of homozygous familial hypercholesterolemia (HoFH). With untreated low-density lipoprotein-cholesterol (LDL-C) at 541 mg/dL, widespread cutaneous and tendon xanthomas, bilateral corneal arcus, and successful percutaneous coronary intervention (PCI) to the left main into the left anterior descending, this report underscores how congenitally toxic LDL levels drive accelerated atherosclerosis at warp speed.

HoFH, biallelic semidominant disorder of the LDL receptor pathway genes, imposes a profound global burden. Prevalence estimates place HoFH at approximately 1 in 300,000 individuals worldwide, translating to roughly 23,000 to 30,000 cases globally, with higher rates in regions of consanguinity or founder effects. Untreated, patients face markedly elevated LDL-C (>400 mg/dL) from birth, leading to premature atherosclerotic cardiovascular disease (ASCVD), aortic valve stenosis, coronary ostial stenosis, supravalvular aortic stenosis, and early mortality—often in the first or second decade of life. Real-world cohorts reveal high ASCVD prevalence, suboptimal LDL-C control despite therapies, and persistent premature death risk, highlighting HoFH as one of the most aggressive inherited lipid disorders.

Central to this condition is the global under-recognition and treatment of HoFH.^2^^,^^3^ Diagnosis and treatment are frequently delayed, often occurring only after manifest ASCVD events such as myocardial infarction or stroke, as seen here where xanthomas were noted for years but untreated due to socioeconomic barriers and limited health care access. Globally, HoFH remains vastly underdiagnosed, with many cases missed in childhood despite visible stigmata. Pediatric lipid screening guidelines are underused, even in high-resource settings, contributing to missed opportunities for early intervention. In low- and middle-income regions, diagnostic delays are compounded by resource limitations, perpetuating cycles of preventable premature events.

The devastating phenotypic expression stems from the undeniable toxic LDL-C exposure from birth. In HoFH, lifelong extreme hypercholesterolemia accelerates plaque formation, endothelial dysfunction, and vascular calcification at an unprecedented pace.^3^ HoFH is atherosclerosis in “warp speed.” Unlike typical ASCVD, which evolves over decades, HoFH can manifest obstructive coronary lesions in childhood or adolescence, as evidenced by the patient in this case report, severe left main bifurcation, and proximal left anterior descending/right coronary artery disease requiring complex PCI. Xanthomas serve as a cutaneous harbinger; however, without prompt recognition, damage ensues arterial plaque formation, potential valvular progression, and heightened sudden death risk. This accelerated trajectory contrasts sharply with heterozygous FH, emphasizing HoFH's unique urgency.

This case highlights actionable imperatives: universal pediatric lipid screening, heightened clinician awareness of physical signs, cascade family screening, and early aggressive lipid-lowering (statins, ezetimibe, PCSK9 inhibitors, apheresis, or emerging ANGPTL3 inhibitors). PCI proved feasible here, but prevention remains paramount. By confronting under-recognition and the relentless burden of untreated HoFH, we can avert such catastrophic presentations and improve survival.

## Funding Support and Author Disclosures

The author has reported that they have no relationships relevant to the contents of this paper to disclose.

## References

[1] Siddiqui F.I., Ghaffar A., Rehman I.U. (2026). Early-onset myocardial infarction in a 13-year-old with homozygous familial hypercholesterolemia managed by PCI. JACC Case Rep.

[2] Cuchel M., Lee P.C., Hudgins L.C. (2023). Contemporary homozygous familial hypercholesterolemia in the United States: insights from the CASCADE FH registry. J Am Heart Assoc.

[3] Cuchel M., Raal F.J., Hegele R.A. (2023). 2023 update on European Atherosclerosis Society Consensus statement on homozygous familial hypercholesterolaemia: new treatments and clinical guidance. Eur Heart J.

